# RAGE and tobacco smoke: insights into modeling chronic obstructive pulmonary disease

**DOI:** 10.3389/fphys.2012.00301

**Published:** 2012-07-25

**Authors:** Adam B. Robinson, Jeffrey A. Stogsdill, Joshua B. Lewis, Tyler T. Wood, Paul R. Reynolds

**Affiliations:** Department of Physiology and Developmental Biology, Brigham Young UniversityProvo, UT, USA

**Keywords:** RAGE, COPD, tobacco, mouse model

## Abstract

Chronic obstructive pulmonary disease (COPD) is a progressive condition characterized by chronic airway inflammation and airspace remodeling, leading to airflow limitation that is not completely reversible. Smoking is the leading risk factor for compromised lung function stemming from COPD pathogenesis. First- and second-hand cigarette smoke contain thousands of constituents, including several carcinogens and cytotoxic chemicals that orchestrate chronic lung inflammation and destructive alveolar remodeling. Receptors for advanced glycation end-products (RAGE) are multi-ligand cell surface receptors primarily expressed by diverse lung cells. RAGE expression increases following cigarette smoke exposure and expression is elevated in the lungs of patients with COPD. RAGE is responsible in part for inducing pro-inflammatory signaling pathways that culminate in expression and secretion of several cytokines, chemokines, enzymes, and other mediators. In the current review, new transgenic mouse models that conditionally over-express RAGE in pulmonary epithelium are discussed. When RAGE is over-expressed throughout embryogenesis, apoptosis in the peripheral lung causes severe lung hypoplasia. Interestingly, apoptosis in RAGE transgenic mice occurs via conserved apoptotic pathways also known to function in advanced stages of COPD. RAGE over-expression in the adult lung models features of COPD including pronounced inflammation and loss of parenchymal tissue. Understanding the biological contributions of RAGE during cigarette smoke-induced inflammation may provide critically important insight into the pathology of COPD.

## Introduction

Chronic obstructive pulmonary disease (COPD) is defined by airflow obstruction that is not fully reversible (Carp and Janoff, 1978). In particular, COPD involves chronic airway inflammation and pulmonary emphysema, which is defined anatomically via pathology samples as an abnormal permanent enlargement of airspaces distal to the terminal bronchioles accompanied by destruction of their walls without obvious fibrosis (Pauwels et al., 2001). COPD morbidity and mortality continue to rise as physician diagnoses of COPD increased from approximately 7 million in 1980 to approximately 13.1 million in 2004 (Adams and Barnes, 2006). COPD was responsible for 8 million outpatient visits, 1.5 million emergency room visits, and 672,000 hospitalizations in the U.S. in 2006 (US Department of Health and Human Services, 2009) and compared to 1980, deaths in 2007 increased 74% to over 124,000 people (American Lung Association COPD Fact Sheet, 2011). While as recent as 2010 the cost associated with COPD was $49.9 billion (Dalal et al., 2010), the precise pathobiochemical basis of COPD exacerbated by voluntary or involuntary tobacco smoke exposure remains enigmatic.

Cigarette smoking is currently the most considerable risk factor for the development of COPD, consisting of emphysema and chronic obstructive bronchitis (Anderson et al., 1964; Fletcher and Peto, 1977; Thun et al., 2000; Hogg, 2004). Notwithstanding, only one quarter of cigarette smokers develop clinically detectible airflow limitation and other symptoms of COPD, suggesting an important role for genetic susceptibility (Sethi and Rochester, 2000; Stockley et al., 2009). Although most people that develop COPD currently smoke cigarettes or have smoked in the past, COPD also develops in individuals that have never smoked (Higgins, 1991). This harmful outcome is due in part to exposure to second-hand smoke (Janson, 2004; Wakefield et al., 2005; Eisner et al., 2006). Furthermore, because some former smokers still live with active smokers and are observed to develop COPD later in life, passive smoke exposure is likely to contribute to disease progression.

First- and second-hand smokers diagnosed with moderate COPD have altered expression of several genes, including transcription factors, growth factors, and extracellular matrix proteins (Ning et al., 2004). These and other gene products likely function to stimulate the recruitment of inflammatory cells, cytokine secretion, cell death, and elevated protease production observed after prolonged cigarette smoke exposure (Carp and Janoff, 1978; Wright and Churg, 1990; Kuschner et al., 1996; Hautamaki et al., 1997; Sopori, 2002). As such, it is critical to examine how genes influence disease presentation so that precise mechanisms through which passive and active cigarette smoke contribute to COPD/emphysema can be identified.

## General mechanisms of COPD pathogenesis

Numerous reviews that address COPD pathogenesis, its impact, and plausible therapies have been composed (Bridevaux and Rochat, 2011; Budinger and Mutlu, 2011; Caramori et al., 2011; Lugade et al., 2011; Rooney and Sethi, 2011). The intent of the current work is to concisely provide a foundational summary of conserved COPD modalities and discuss the plausible influence of receptors for advanced glycation end-products (RAGE) signaling. The prevailing pathogenic concept states that COPD is associated with chronic inflammation, imbalances between proteases/antiproteases, oxidative stress, and an elevated apoptotic index. Inflammation arising predominantly from neutrophilic contributions has been proposed due to enhanced neutrophil abundance in bronchoalveolar lavage (BAL) and sputum from COPD patients (Thompson et al., 1989; Stanescu et al., 1996; O'Donnell et al., 2004). Levels of chemoattractants that recruit neutrophils and other potent inflammatory mediators are also elevated in COPD, including leukotriene B4 (Beeh et al., 2003), CXCL2 and 8 (Keatings et al., 1996; Tanino et al., 2002; Beeh et al., 2003), CXCL1 (Keatings et al., 1996), CXCL5 (Tanino et al., 2002), IFN-γ (Hodge et al., 2007), IL-1β (Thacker, 2006; Churg et al., 2009), and TNF-α (Barnes and Karin, 1997). Matrix metalloproteinases (MMPs) produced by macrophages and neutrophils are also misregulated in COPD (Shapiro, 1994). In particular, levels of MMP-1, MMP-2, MMP-7, MMP-9, and MMP-12 are all up-regulated in pulmonary tissue, BAL, and/or sputum of patients with COPD (Shapiro et al., 1993; Hautamaki et al., 1997; Ohnishi et al., 1998; Pratico et al., 1998; Shaykhiev et al., 2009), however because smoke exposed MMP-9 knockout mice are protected from emphysema, MMP-9 may require cooperation with other proteases during adverse lung remodeling (Atkinson et al., 2011) The chemical assessment of tobacco smoke reveals that it contains high levels of reactive oxygen species (ROS) that are in excess of intrinsic antioxidant defense mechanisms (Pauwels et al., 2001; Barnes et al., 2003). Generated in the airways, oxidants lead to cell dysfunction and/or death and also influence inflammatory signaling and protease augmentation via NF-κB-mediated mechanisms (Moodie et al., 2004). During the last decade, enhanced apoptosis stemming from diverse signaling pathways has also been implicated in alveolar septal cell loss observed in COPD patients (Kasahara et al., 2000, 2001; Tuder et al., 2003; Petrache et al., 2006). As a programmed event of removing unwanted cells and debris, apoptosis occurs via extrinsic signaling processes (Degterev et al., 2003), and intrinsic mitochondria or endoplasmic reticulum-mediated processes (Darmon et al., 1995; Slee et al., 1999). In summary, COPD is characterized by progressive destruction of the distal lung and small airway obstruction resulting from chronic inflammation and elevated cell death.

## Constituents of tobacco smoke

Tobacco smoke is a toxic and carcinogenic mixture of more than 5000 chemicals (Talhout et al., 2011). Of these, around 400 have been quantified, at least 200 are toxic to humans and/or experimental animals, and over 50 have been identified as known, probable, or possible human carcinogens (Kirsti, 2004). Studies indicate that compared with mainstream smoke collected under standard FTC/ISO smoking parameters, sidestream smoke has higher levels of PAHs (Grimmer et al., 1987; Evans et al., 1993), nitrosamines (Brunnemann et al., 1977, 1980; Hoffmann et al., 1979a; Ruhl et al., 1980), aza-arenes (Dong et al., 1978; Grimmer et al., 1987), aromatic amines (Patrianakos and Hoffmann, 1979), carbon monoxide (Hoffmann et al., 1979b; Rickert et al., 1984), nicotine (Rickert et al., 1984; Pakhale and Maru, 1998), ammonia (Brunnemann and Hoffmann, 1975), pyridine (Johnson et al., 1973; Brunnemann and Hoffmann, 1978), and the gas phase components 1,3-butadiene, acrolein, isoprene, benzene, and toluene (Brunnemann et al., 1990). In addition to these deleterious compounds, other factors such as the type of tobacco, the chemicals added to the tobacco, the way the tobacco product is smoked, and, for cigarettes and cigars, the material in which the tobacco is wrapped can also affect second-hand smoke chemical composition (International Agency for Research on Cancer, 2002; National Toxicology Program, 2005; US Department of Health and Human Services, 2006).

Cigarette smoke is also an important exogenous source of reactive glycation products capable of promoting formation of AGEs, advanced glycation end-products, which are irreversibly glycated proteins that efficiently bind RAGE (Cerami et al., 1997). Studies have shown that both aqueous extracts of tobacco and cigarette smoke contain glycotoxins, highly reactive glycation products that can rapidly induce AGE formation on proteins *in vitro* and *in vivo* (Nicholl and Bucala, 1998; Nicholl et al., 1998). These activities can be eliminated by passing the samples through a dry packed column of aminoguanidine, a potent and specific inhibitor of AGE formation. Additional studies have shown that serum AGEs and apolipoprotein B-linked AGE levels are significantly elevated in cigarette smokers relative to non-smokers and AGEs or immunochemically related molecules are present at higher levels in the tissues of smokers compared to non-smokers, regardless of the presence of diabetes (Nicholl et al., 1998).

## Receptor for advanced glycation end-products

**Figure 1:**
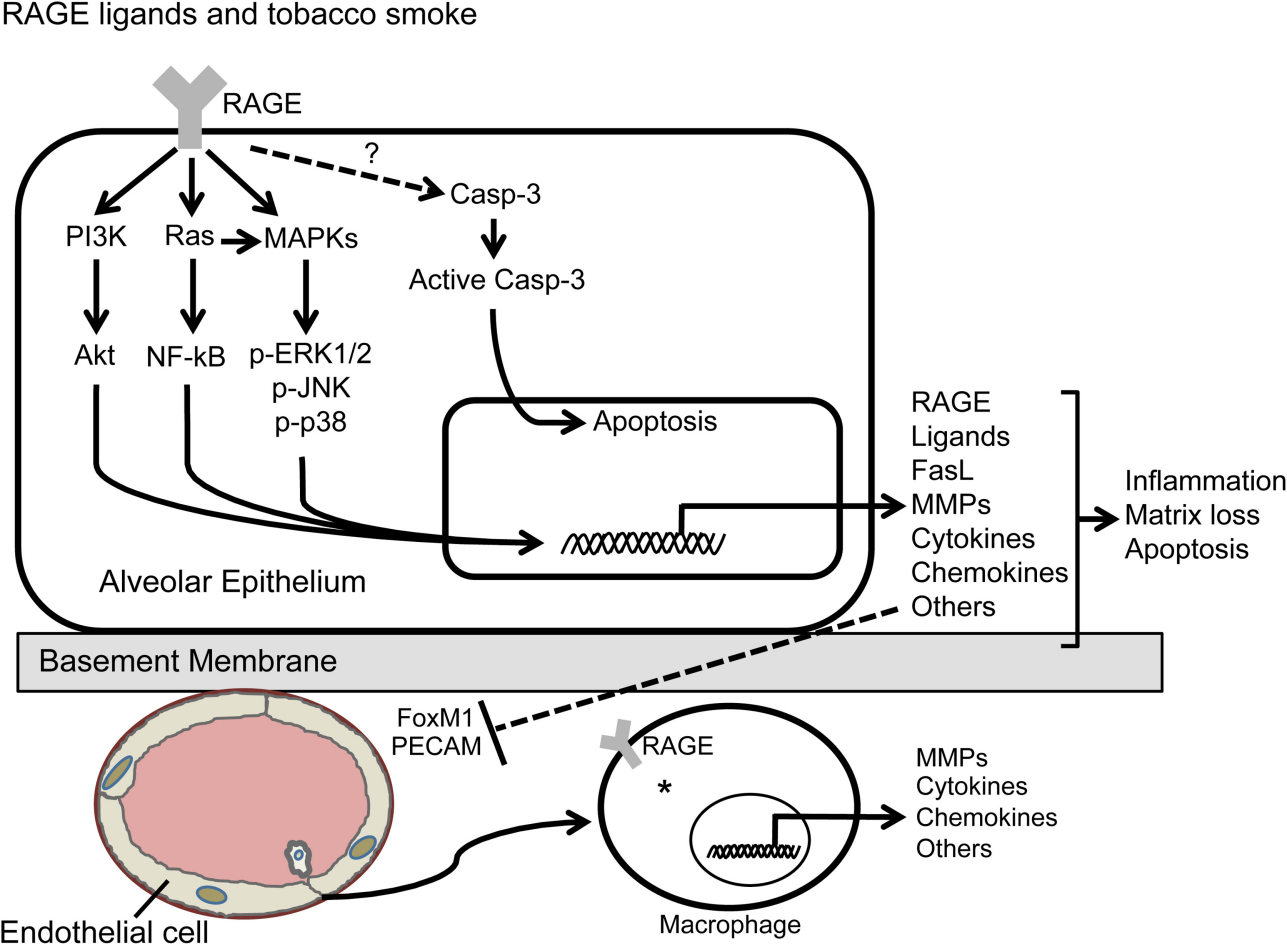
Deleterious effects characteristic of COPD are elicited via several pro-inflammatory signaling pathways observed in RAGE-expressing alveolar epithelial cells and resident alveolar macrophages (*). Direct stimulation of RAGE by tobacco smoke, *de novo* AGE generation in a tobacco smoke environment, or genetic up-regulation of RAGE in the lungs of conditional bi-transgenic mice results in characteristics of COPD including inflammation, matrix destabilization, and apoptosis.

RAGE are cell-surface receptors of the immunoglobulin superfamily expressed in many cell types including endothelial and vascular smooth muscle cells, fibroblasts, macrophages/monocytes, and epithelium (Brett et al., 1993). RAGE expression is most abundant in the lung, from which it was initially isolated, and is selectively localized to well-differentiated alveolar type I (ATI) epithelial cells (Schmidt, 2001). Identification in respiratory epithelium (Dahlin et al., 2004; Koslowski et al., 2004) and studies that document RAGE-mediated adherence to collagen IV (Demling et al., 2006) have led to the implication of RAGE in important developmental processes such as the spreading, thinning, and adherence that characterize the transitioning of ATII cells to squamous ATI cells. RAGE was first described as a progression factor in cellular responses induced by AGEs that accumulate in hyperglycemia and oxidant stress. Subsequent studies have distinguished RAGE as a pattern recognition receptor that also binds S100/calgranulins, amyloid-β-peptide, and HMGB-1 (or amphoterin), to influence gene expression via divergent signal transduction pathways (Reddy et al., 2006; Hudson et al., 2008; Kim et al., 2008; Toure et al., 2008). Because RAGE expression can also increase when ligands accumulate (Schmidt, 2001), RAGE-ligand interaction may contribute to chronic pathological states where ligands are common including diabetic complications, neurodegenerative disorders, atherosclerosis, and inflammation (Hofmann et al., 1999; Taguchi et al., 2000). Specifically, a host of pro-inflammatory responses such as those coordinated by MAP kinases (ERK, JNK, and p38), NF-κB, ROS, and other pro-inflammatory mediators such as TNF and IL-1 (Bianchi et al., 2010) result from RAGE-ligand interactions (Figure 1). In contrast to short-lived cellular activation mediated by LPS, engagement of RAGE by its ligands results in prolonged inflammation (Lin et al., 2009). If left unchecked, such chronic inflammation results in severe tissue injury.

The full length membrane bound form of RAGE (mRAGE) contains an extracellular variable V-region-like immunoglobulin domain crucial for ligand binding and two constant C-region-like immunoglobulin domains, a single-pass hydrophobic transmembrane domain and a short, 43 amino acid, highly charged cytoplasmic domain essential for intracellular signaling (Buckley and Ehrhardt, 2010). The cytoplasmic domain of RAGE contains four possible phosphorylation sites, S391, S399, S400, and T401, of which only S391 is conserved among humans, mice, guinea pigs, rats, rabbits, dogs, and cats (Sakaguchi et al., 2011). Replacement of S391 to alanine was sufficient to abrogate PKCζ-dependent phosphorylation and subsequent signal transduction *in vitro* (Sakaguchi et al., 2011). Although not explicitly stated, RAGE behaves similarly to a receptor tyrosine kinase (RTK) cell surface receptor, requiring homodimerization to effectively potentiate intracellular signaling cascades (Zong et al., 2010). Distinct alternative isoforms also exist for the receptor due to differential splicing variants of the RAGE message. Dominant negative RAGE (dn-RAGE) is a membrane anchored splice variant of RAGE capable of ligand binding but lacking the intracellular domain necessary for signal transduction. Endogenous secreted RAGE (esRAGE) is generated via alternative splicing at exon 9 yielding the same V and C-regions of the full length-RAGE but lacks both the hydrophobic transmembrane and the intracellular domains (Buckley and Ehrhardt, 2010). Additionally, full-length RAGE can be cleaved by MMPs to render sRAGE, a non-splice variant of RAGE closely resembling esRAGE in structure and function (Yamakawa et al., 2011). These altered variants of RAGE incapable of transducing signals are thought to function as decoy receptors that prevent the interaction of mRAGE with its ligands.

The pro-inflammatory role of RAGE in cardiovascular diseases is well documented (Yan et al., 2009). Furthermore, several studies strongly suggest that RAGE signaling is a key regulator of inflammation in major pulmonary diseases. A study demonstrated that abrogation of RAGE signaling (using RAGE null mice) attenuated pulmonary ischemia and reperfusion injury associated with decreased NF-κB activation and IL-8 production (Sternberg et al., 2008). Another important role for RAGE signaling in lung disease shows that RAGE-deficient mice under hyperoxic conditions survived longer than wild type controls and the mice had less airway cellularity and diminished alveolar damage compared to wild type controls (Reynolds et al., 2010). RAGE has been implicated in the fibrotic process in a number of tissues, including the peritoneum, kidney, and liver (Li et al., 2004; De Vriese et al., 2006; Xia et al., 2008), where it has been shown to promote fibrosis. In the lung, evidence continues to accumulate suggesting an important role for RAGE in pulmonary fibrosis, yet conflicting data portray RAGE as having both protective and destabilizing functions. Acute lung injury (ALI) and a more severe condition known as acute respiratory distress syndrome (ARDS) are characterized by deterioration of the alveolar-capillary barrier and impaired alveolar fluid clearance (Lucas et al., 2009). ALI and ARDS are associated with damage to ATI cells, a population of cells with significant RAGE expression, and several different animal models of ALI express increased RAGE levels in BALF (Uchida et al., 2006; Su et al., 2007, 2009; Zhang et al., 2008). A published study from our laboratory considered the effects of smoke exposure on RAGE expression both in lung cells and mice (Reynolds et al., 2008). The research revealed that RAGE and its ligands were up-regulated in lung epithelial cells cultured with cigarette smoke extract (CSE) and that mice exposed to cigarette smoke for 6 months had elevated RAGE expression in pulmonary epithelium (Reynolds et al., 2008). While the full extent of RAGE function in smoke-induced COPD has not been sufficiently examined, these studies demonstrate that RAGE may play a role in COPD pathogenesis.

## Contributions of rage to COPD progression

RAGE and two of its ligands S100A12 and HMGB-1 were up-regulated in a rat alveolar type I-like cell line (R3/1), a human alveolar type 1I-like epithelial cell line (A549), and a macrophage-like murine cell line (RAW 264.7) following exposure to CSE (Reynolds et al., 2008). S100A12 is a calcium-binding pro-inflammatory modulator and HMGB-1 is a non-histone nuclear protein that acts as a potent pro-inflammatory mediator when secreted. In human lungs with smoke-related lesions, widespread RAGE expression has been documented in bronchiolar epithelia, small respiratory airways, reactive ATI cells, and alveolar macrophages (AMs; Morbini et al., 2006). The same study identified elevated S100A12 in polymorphonuclear granulocytes and in extracellular fluid and the number and intensity of carboxymethyl-lysine positive cells (cells that stain for AGEs) were measurably enhanced in epithelial and inflammatory cells of the lungs of smokers (Morbini et al., 2006).

Another factor highly expressed in the lungs of smokers with COPD is early growth response gene 1 (Egr-1), a zinc finger-containing, hypoxia-inducible transcription factor (Ning et al., 2004). Egr-1 expression significantly increased in lung cell lines following CSE exposure *in vitro* and it activated the RAGE promoter (Reynolds et al., 2006, 2008). Because the RAGE gene also contains NF-κB and SP-1 promoter response elements (Li and Schmidt, 1997) and is transcriptionally regulated by cis-acting Egr-1 (Reynolds et al., 2006), a possible auto-inflammatory loop may be triggered suggesting cooperation between Egr-1 and RAGE in chronic smoke-related inflammatory disease states. More recently, it was discovered that Ras, a small GTPase that functions as a molecular switch in the control of diverse signaling cascades, was induced in R3/1 cells following exposure to CSE, resulting in up-regulation of NF-κB-mediated secretion of TNF-α, IL-1β, and IL-8 (Figure 1; Reynolds et al., 2011a).

Our lab has recently expanded research into the biology of smoke-exposed primary mouse AMs also known to express RAGE. Studies document that low levels of RAGE are expressed by mouse primary macrophages during normal conditions and that RAGE overexpression by these primary macrophages is associated with inflammation and the coordination of lung damage (Morbini et al., 2006). Our studies indicate that acute exposure of mice to CSE via nasal instillation resulted in diminished BAL cellularity and fewer AMs in RAGE null mice compared to controls. Additionally, AMs isolated from wild type mice exposed to CSE significantly increased RAGE expression (Robinson et al., 2012). This recently published work also demonstrated for the first time that RAGE null AMs exposed to CSE experienced reduced Ras and p38 MAPK activation, less NF-κB translocation, and diminished expression of TNF-α and IL-1β when compared to CSE exposed wild type AMs (Figure 1). Evidence suggests that primary AMs coordinate CSE-induced inflammation, at least in part, via RAGE-mediated mechanisms and that cooperation with alveolar epithelium in coordinated inflammatory responses is likely.

## Use of rage transgenic mice in modeling characteristics of COPD

Several animal models that seek to recapitulate various aspects of COPD have been presented within the past decade. These models include mouse IL-1β over-expressers (Lappalainen et al., 2005), rat VEGF signaling nulls (VEGF or VEGFR2 blockers: Kasahara et al., 2000), intratracheal administration of active caspase-3 (Aoshiba et al., 2003) and several others that aim to elucidate inflammatory and other destructive mechanisms during smoke-less and smoke-exposed disease progression (Petrache et al., 2005; Giordano et al., 2008; Kang et al., 2012). The vast majority of these models present emphysema-like anatomical characteristics and inflammatory indexes in the presence of room-air and notable exacerbation in the presence of cigarette smoke. Although RAGE has been shown to be a marker for many inflammatory diseases including COPD, a genetic mouse model for COPD had not been previously examined.

**Figure 2:**
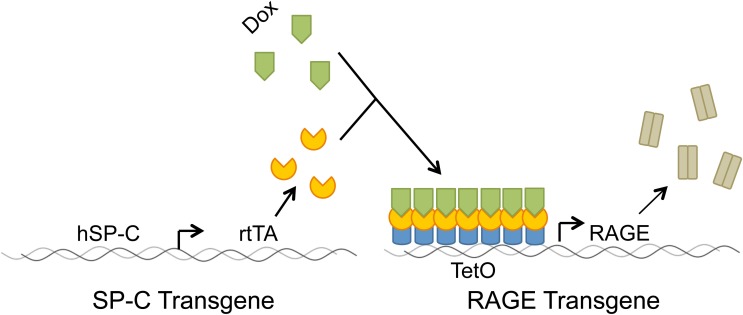
**Full length RAGE was over-expressed in alveolar type (AT) II cells by obtaining progeny from two transgenic lines of mice.** The reverse tetracycline transactivator (rtTA) was produced under the control of the human surfactant protein C (hSP-C) promoter in distal respiratory epithelium. Following the administration of doxycycline (dox), a dox-rtTA complex activates the TetO promoter, thereby expressing RAGE.

We generated a bi-transgenic *in vivo* mouse model that utilizes two transgenes to conditionally up-regulate RAGE (Figure 2). One transgenic mouse line employs surfactant protein C (SP-C) to drive expression of rtTA (reverse tetracycline transactivator) and another transgenic line contains binding sites for a complex between rtTA and doxycycline (dox; Reynolds et al., 2011b). Although COPD is an adult lung disease, we initially sought to characterize RAGE bi-transgenic mice during development with the realization that aspects of COPD may be detected during organogenesis. Our model was thought to compliment research that centers on bronchopulmonary dysplasia (BPD), an embryonic disease highly correlated with emphysema in terms of oxidative stress, pulmonary inflammation, increased apoptosis, protease/antiprotease imbalance and altered microvasculature (Hargitai et al., 2001; Danan et al., 2002; Saugstad, 2003; Ekekezie et al., 2004; Speer, 2006). While COPD is characterized by sustained inflammation and alveolar destruction, remarkably similar mechanisms are implicated in the altered branching and impaired alveolarization observed in BPD (Bourbon et al., 2009).

### Embryonic rage bi-transgenic mice have perturbed distal epithelium

Complete perinatal lethality was observed when dox was supplied to RAGE bi-transgenic mice throughout embryogenesis. At embryonic day (E) 18.5, pulmonary tissues were severely hypoplastic and only minimal respiratory surface area near the visceral pleura remained. Several immunohistochemical and flow cytometric experiments demonstrated diminished abundance of differentiated distal lung cell types, most notably ATI and ATII cells (Reynolds et al., 2011b).

Altered cellular differentiation has not sufficiently been characterized in the distal lung of COPD patients; however, new research has emerged demonstrating that human ciliated cells can respond to cigarette smoke by promoting GDF15, a factor capable of driving Muc5A expression in goblet cells (Wu et al., 2011). RAGE and RAGE ligands have been implicated in altered cellular differentiation of several cell types including smooth muscle cells, skeletal myocytes and developing neural tissue (Suga et al., 2011; Kim et al., 2012; Riuzzi et al., 2012). Thyroid transcription factor 1 (TTF-1; also known as Nkx2.1) is a key regulator of pulmonary development and present in distal lung epithelium that can negatively regulate RAGE expression (Reynolds et al., 2008) and SP-1 positively regulates the active promoter region of TTF-1 in surfactant producing cells (Das et al., 2011). Because NF-κB (a crucial intermediate of RAGE signaling) can interfere with SP-1 binding (Benjamin et al., 2010), RAGE may play a role in inhibited surfactant synthesis observed when ATII cells are abnormally regulated.

### Embryonic rage bi-transgenic mice have abnormal distal pulmonary endothelial cell growth

In addition to the decreased cellularity of the lungs, RAGE over-production disturbed capillary growth and maintenance through the inhibition of FoxM1 (a critical transcription factor necessary for endothelial expansion) and PECAM (a marker for endothelial cells) expression (Geyer et al., 2011). Endothelial cell apoptosis has been observed in COPD patients using TUNEL, immunohistochemistry and DNA ligation techniques that coincided with the reduction of endothelial markers including VEGF and VEGFR2 (Kasahara et al., 2001). Additionally Dinh-Xuan et al. and Peinado et al. both showed that resected lung samples from COPD patients had extensive endothelial dysfunction, which they proposed to contribute to hypertension (Dinh-Xuan et al., 1991; Peinado et al., 1998). It is hypothesized that vascular tone in the lung can be regulated by direct stimulation of the vascular compartment by cigarette smoke and indirect stimulation stemming from smoke-exposed epithelial cells. Our discoveries relating to pulmonary endothelium in the RAGE bi-transgenic mouse correlate with numerous studies that demonstrate RAGE signaling in cases of depressed endothelial function and increased barrier disruption (Sun et al., 2009; Pollreisz et al., 2010; Wolfson et al., 2011; Chen et al., 2012; Huang et al., 2012).

### Embryonic rage bi-transgenic mice have extracellular matrix abnormalities

We also demonstrated that MMP-9 secretion is increased, coincident with diminished collagen IV (a principle component of the alveolar basement membrane) deposition and production (Bukey et al., 2011). COPD is characterized by an increase in several MMPs including MMP-1, 2, 9, and 12 (Ohnishi et al., 1998; Geraghty et al., 2011). Other research groups have also demonstrated AGE-RAGE dependent mechanisms in MMP-9 production (Ishibashi et al., 2010; Zhang et al., 2010; Zhu et al., 2012). While not yet evaluated in our embryonic RAGE bi-transgenic mouse model, MMPs 1 and 2 have been implicated as RAGE targets (Kamioka et al., 2011; Du et al., 2012; Yu et al., 2012). Interestingly, MMP-1 has been shown to be up-regulated not only in the lungs of COPD patients but in osteoarthritis as well, a chronic inflammatory disease affecting articular cartilage (Steenvoorden et al., 2006). Ongoing research seeks to test hypotheses related to matrix-targeting protease imbalances such as those that involve α1-antitrypsin.

### Embryonic rage bi-transgenic mice have elevated parenchymal cell apoptosis

Thorough evaluations of apoptosis were performed in order to ascertain causes for the hypoplastic lung phenotype in the embryonic RAGE bi-transgenic mouse. RAGE over-expressing lungs detrimentally declined during the canalicular phase, a period identified by terminal bronchiole branching, initial alveolarization, and microvascular organization. The abrupt loss of tissue was observed in tandem with a significant increase in pro-apoptotic Fas ligand (FasL), a decrease in the anti-apoptotic factor Bcl-2, elevated cleaved active caspase-3 (a critical mediator of cell death), and quantifiable apoptosis by TUNEL assessment (Stogsdill et al., 2012). Electron microscopy also confirmed apoptosis via the detection of numerous bleb-like structures within cells that were physically separated from the underlying basement membrane. Importantly, cellular proliferation was not changed, suggesting there was no feedback mechanism to compensate for elevated cell death. Evidence is mounting that demonstrates active apoptosis of epithelial and endothelial cells in human COPD patients (Segura-Valdez et al., 2000; Kasahara et al., 2001; Majo et al., 2001; Yokohori et al., 2004; Hodge et al., 2005; Imai et al., 2005). Lending support for FasL-mediated apoptosis observed in RAGE bi-transgenic mice was research by Mahali et al. that demonstrated FasL elaboration is a direct product of AGE-RAGE ligation (Mahali et al., 2011). Furthermore, RAGE and its ligands have been shown to promote apoptosis in other tissue types, including myocytes (Tsoporis et al., 2010), endothelial cells (Chen et al., 2010), neuronal cells (Kim et al., 2011), epithelial cells (Jin et al., 2011), and pancreatic β-cells (Lee et al., 2010). Our studies have shown for the first time that increased expression of RAGE using transgenic mouse technology directly activates apoptosis in lung parenchyma. In fact, sustained RAGE expression during development is capable of modeling disorders characterized by cell loss including BPD. Furthermore, these data reveal important RAGE-mediated mechanisms that control cell quantity possibly introduced at the initiation of smoke-induced COPD pathogenesis.

### Adult rage over-expression yields an emphysematous lung

Conditional up-regulation of RAGE for 2 to 3 months in the adult bi-transgenic mouse lung lead to incremental dilation of alveolar spaces, assessed by standard H&E staining (Stogsdill et al., 2011). Quantification of the mean chord length of the airspace revealed progressive dilation of alveolar spaces as RAGE over-expression persisted (unpublished data). The adult RAGE bi-transgenic mice had increased MMP-9 and decreased elastin expression consistent with other COPD models. Furthermore, RAGE bi-transgenic mice manifested significant inflammation measured by elevated BALF protein, leukocyte infiltration, and secreted cytokines (MIP-2, IFN-γ; Stogsdill et al., 2011). These data support the concept that innovative transgenic mice that over-express RAGE may model pulmonary inflammation and alveolar destabilization independent of tobacco smoke. Furthermore, it validates RAGE signaling as a target pathway in the prevention or attenuation of smoke-related inflammatory lung diseases.

## Conclusions

Despite the progression in the field of RAGE biology in the context of lung disease, the full extent of RAGE localization, the molecular mechanisms that control its expression and its downstream effects should remain topics of focused investigation. While a great deal is known about COPD, relatively little is known about factors that perpetuate inflammation or modalities that sustain them. Our research has shown that mechanisms of COPD progression including chronic inflammation, imbalances involving proteases, oxidative stress, and elevated apoptosis may be mediated by RAGE. Several endogenous (S100/calgranulins, HMGB-1, AGEs) and exogenous ligands (cigarette smoke) may be responsible for the sustained activation of RAGE leading to disease progression (Figure 1). As such, it remains possible that targeting RAGE may, at least in part, provide successful opportunities in the therapeutic alleviation of debilitating inflammatory lung disease exacerbated by tobacco smoke.

### Conflict of interest statement

The authors declare that the research was conducted in the absence of any commercial or financial relationships that could be construed as a potential conflict of interest.
